# Supplementary material for: Disease-associated gut microbiome and metabolome changes in patients with chronic obstructive pulmonary disease
Source: Nat Commun. 2020 Nov 18;11:5886. doi: 10.1038/s41467-020-19701-0 (PMC7676259; doi:10.1038/s41467-020-19701-0)
Supplement: Supplementary file 1 — Supplementary Information [file 41467_2020_19701_MOESM1_ESM.pdf]

**Supplementary material**

# Disease-associated gut microbiome and metabolome changes in patients with chronic obstructive pulmonary disease

Kate L Bowerman[1+], Saima Firdous Rehman[2+], Annalicia Vaughan[3], Nancy Lachner[1], Kurtis F Budden[2], Richard Y Kim[4], David LA Wood[1], Shaan L Gellatly[2], Shakti D Shukla[2], Lisa G. Wood[2], Ian A. Yang[3], Peter A Wark[2#], Philip Hugenholtz[1#], Philip M Hansbro[2,4#*]

[+] Contributed equally

# Contributed equally

[1]Australian Centre for Ecogenomics, School of Chemistry and Molecular Biosciences, The University of Queensland, Queensland, Australia. [2]Priority Research Centre for Healthy Lungs, Hunter Medical Research Institute, New South Wales, Australia & The University of Newcastle, New South Wales, Australia. [3]Thoracic Research Centre, Faculty of Medicine, The University of Queensland, and Department of Thoracic Medicine, The Prince Charles Hospital, Brisbane, Queensland, Australia. [4]Centre for Inflammation, Centenary Institute & University of Technology Sydney, School of Life Sciences, Faculty of Science, Sydney, New South Wales, Australia.

Correspondence and requests for materials should be addressed to P.M.H (email: philip.hansbro@uts.edu.au)

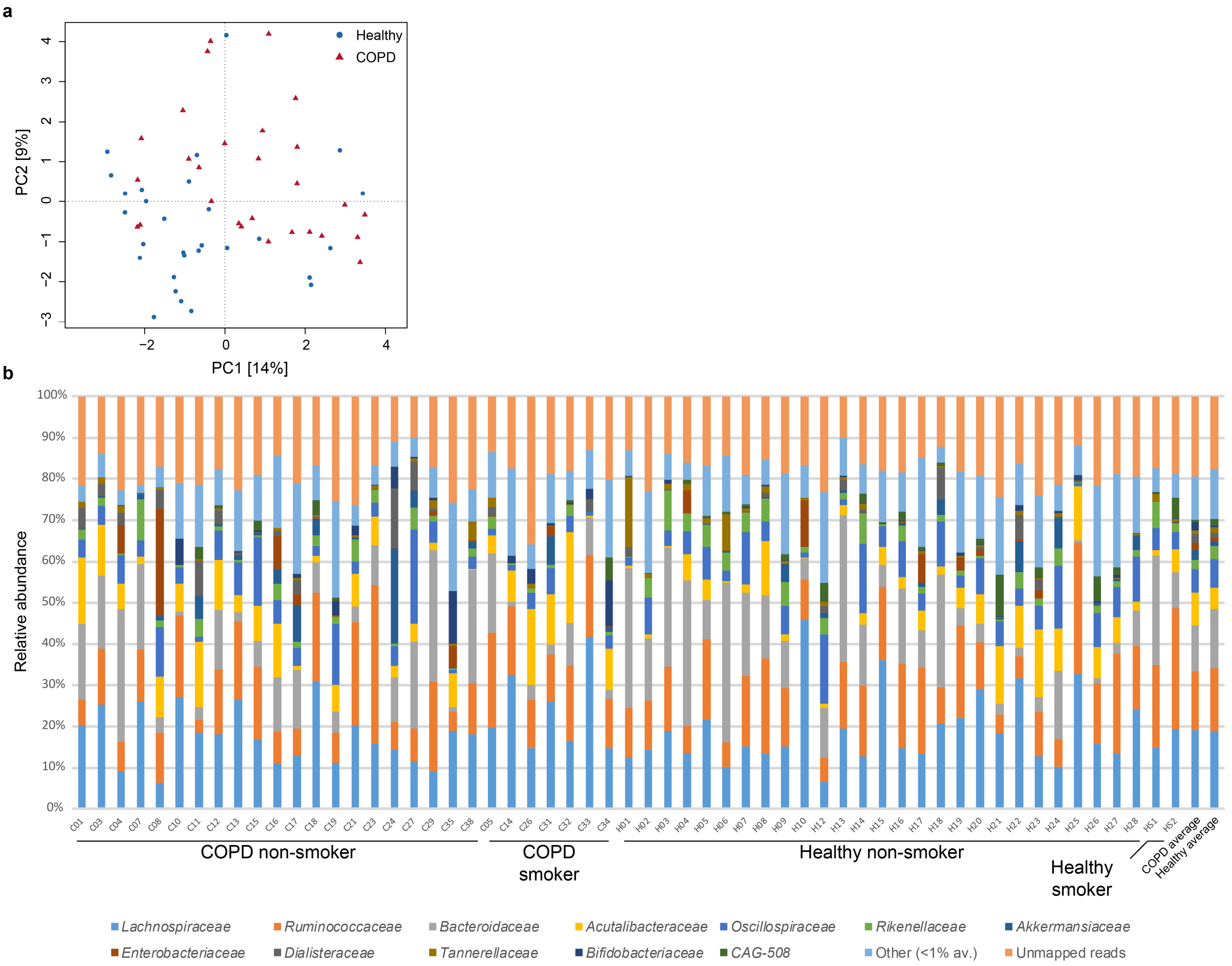


**Supplementary Fig. 1** Metagenome sequencing identifies COPD-associated faecal microbiota. **a** Principal component analysis at the genome level based on read count mapping to each genome. **b** Read counts were transformed prior to analysis using log cumulative-sum-scaling. Family level relative abundance presented. Each bar represents an individual sample (n = 57, COPD: n = 28; healthy: n = 29.). Families with average relative abundance <1% are grouped under Other. Unmapped indicates percentage of reads that did not map to the genome database. **a** and **b** are based on read counts per genome generated from read mapping to recovered metagenome-assembled genomes plus reference genomes available from NCBI.

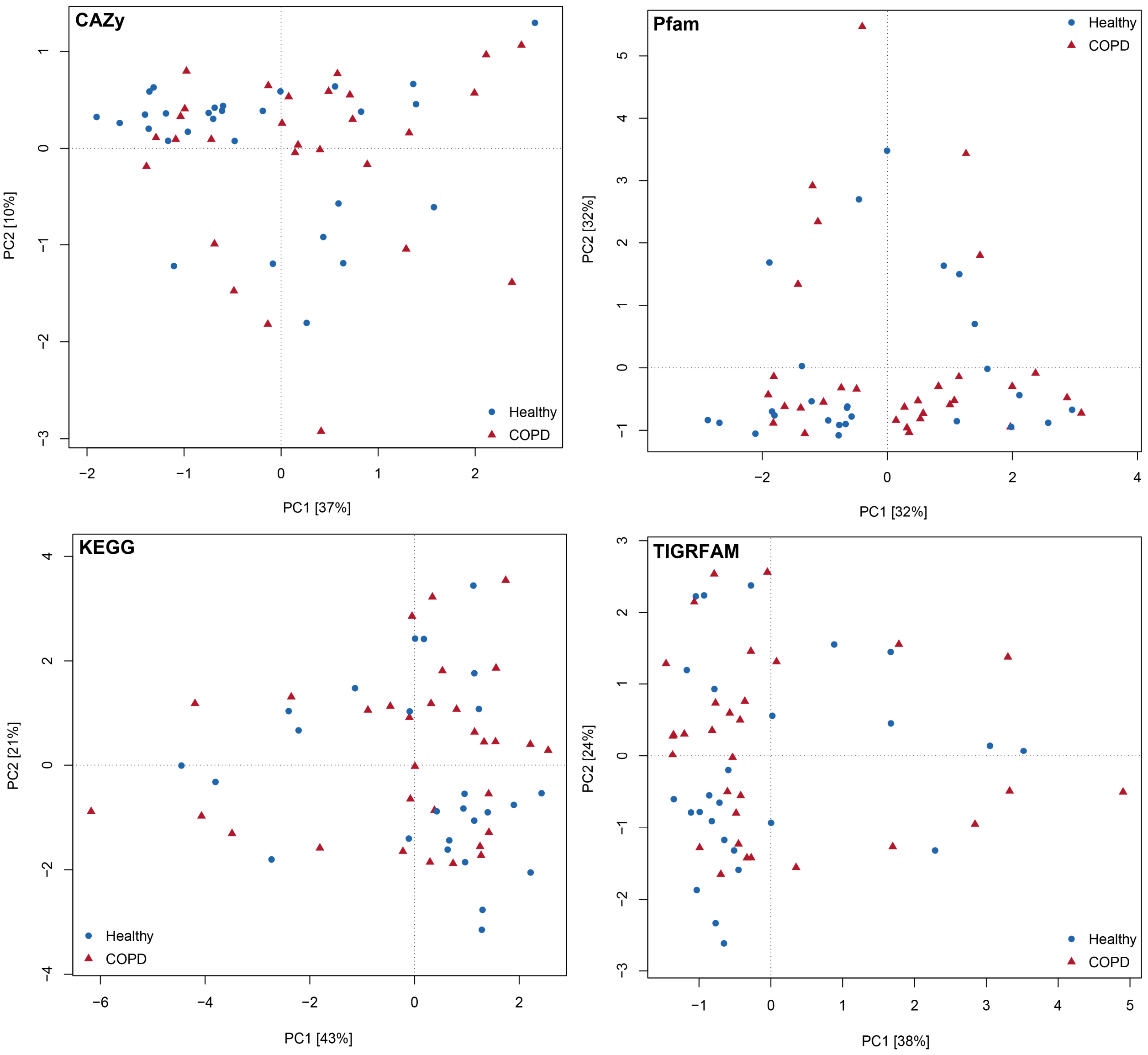


**Supplementary Fig. 2** Predicted functional capacity of gut metagenome does not distinguish between COPD and healthy samples at the global level. Principal component analysis of read counts per domain following annotation of raw reads with predicted function based on alignment with Pfam, TIGRFAM, KEGG and CAZy databases. No significant difference was identified between COPD and healthy samples: PERMANOVA $P_{pfam}$=0.5117; $P_{tigrfam}$=0.5688; $P_{kegg}$=0.5616; $P_{cazy}$=0.1247. COPD: n = 28; healthy: n = 29.

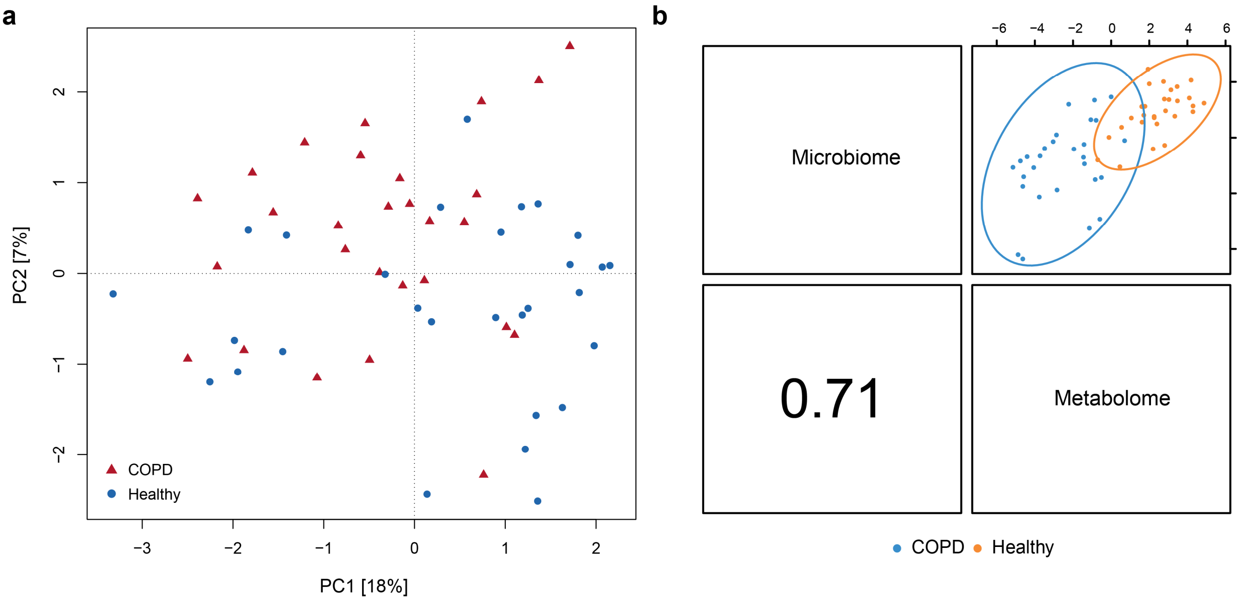


**Supplementary Fig. 3** Gut metabolome of COPD patients is distinct from healthy individuals and correlated with the gut microbiome. **a** Principal component analysis of log-transformed median-scaled metabolomic data. PERMANOVA P=0.003. **b** MixOmics DIABLO correlation plot displaying Pearson correlations between metagenomic and metabolomic data on the first principal component. The correlation between the two data types supports their integration and the presentation a joint signature. COPD: n = 28; healthy: n = 29.
