# Supplementary material for: Disease-associated gut microbiome and metabolome changes in patients with chronic obstructive pulmonary disease
Source: Nat Commun. 2020 Nov 18;11:5886. doi: 10.1038/s41467-020-19701-0 (PMC7676259; doi:10.1038/s41467-020-19701-0)
Supplement: Supplementary file 3 — Reporting Summary [file 41467_2020_19701_MOESM3_ESM.pdf]

nature research

Corresponding author(s): Prof.Philip M Hansbro

Last updated by author(s): Oct 16, 2020

Reporting Summary

Nature Research wishes to improve the reproducibility of the work that we publish. This form provides structure for consistency and transparency in reporting. For further information on Nature Research policies, see our Editorial Policies and the Editorial Policy Checklist.

Statistics

For all statistical analyses, confirm that the following items are present in the figure legend, table legend, main text, or Methods section.

n/a	Confirmed	
☐	☒	The exact sample size (*n*) for each experimental group/condition, given as a discrete number and unit of measurement
☐	☒	A statement on whether measurements were taken from distinct samples or whether the same sample was measured repeatedly
☐	☒	The statistical test(s) used AND whether they are one- or two-sided<br>*Only common tests should be described solely by name; describe more complex techniques in the Methods section.*
☐	☒	A description of all covariates tested
☐	☒	A description of any assumptions or corrections, such as tests of normality and adjustment for multiple comparisons
☐	☒	A full description of the statistical parameters including central tendency (e.g. means) or other basic estimates (e.g. regression coefficient) AND variation (e.g. standard deviation) or associated estimates of uncertainty (e.g. confidence intervals)
☐	☒	For null hypothesis testing, the test statistic (e.g. *F*, *t*, *r*) with confidence intervals, effect sizes, degrees of freedom and *P* value noted<br>*Give P values as exact values whenever suitable.*
☒	☐	For Bayesian analysis, information on the choice of priors and Markov chain Monte Carlo settings
☒	☐	For hierarchical and complex designs, identification of the appropriate level for tests and full reporting of outcomes
☐	☒	Estimates of effect sizes (e.g. Cohen's *d*, Pearson's *r*), indicating how they were calculated

Our web collection on statistics for biologists contains articles on many of the points above.

Software and code

Policy information about availability of computer code

Data collection	Sequencing data produced using the Illumina platform (MiSeq & NextSeq500), metabolomics data produced by Metabolon Inc. using the Metabolon HD4 platform using database servers operating on Oracle 10.2.0.1 Enterprise Edition.
Data analysis	Open source software: Cutadapt v1.1, Trimmomatic v0.36, QIIME2 & QIIME v1.8.0, BLAST v2.8.1, BWA v0.7.12, Spades v3.12.0, BamM v1.7.3, Metabat v2.12.1, CheckM v1.0.11, dRep v2.05, GTDB-Tk v0.3.0, Mosdepth v0.2.3, Prodigal v2.6.3, HMMER v3.1b2, EnrichM v0.5.0<br>R packages: DADA2 v1.12, DESeq2 v1.22.2, NormalizeMets v0.25, psych v1.8.12, corrplot v0.84, pheatmap v1.0.12, mixOmics v6.6.2, metagenomeSeq v1.22.0 & v1.24.1, vegan v2.5-1 & v2.5-5

For manuscripts utilizing custom algorithms or software that are central to the research but not yet described in published literature, software must be made available to editors and reviewers. We strongly encourage code deposition in a community repository (e.g. GitHub). See the Nature Research guidelines for submitting code & software for further information.

Data

Policy information about availability of data

All manuscripts must include a data availability statement. This statement should provide the following information, where applicable:

- Accession codes, unique identifiers, or web links for publicly available datasets
- A list of figures that have associated raw data
- A description of any restrictions on data availability

The 16S rRNA amplicon and metagenomic sequencing data have been deposited to the NCBI Sequence Read Archive under accession PRJNA562766 (https://www.ncbi.nlm.nih.gov/sra). Recovered MAGs have been deposited to the NCBI DDBJ/ENA/GenBank database under accessions WGSA00000000-WHIU00000000 (https://www.ncbi.nlm.nih.gov/genbank). Prokka annotated MAG sequences in GenBank format are available at https://github.com/katebowerman/COPD. Sample accessions are provided in Supplementary Tables 28-30. Sequence variant read counts from 16S rRNA amplicon sequencing (raw data underlying figure 1) and

metagenomic genome-based mapping counts (raw data underlying figures 2-7) are provided as a Source Data File. The reference human genome used in this study (Homo_sapiens.GRCh38) is available at https://www.ncbi.nlm.nih.gov/assembly/2334371. Reference bacterial genomes are available from https://www.ncbi.nlm.nih.gov/assembly/. Additional databases used in this study are available as follows: SILVA v132 (https://www.arb-silva.de/no_cache/download/archive/release_132/Exports/), GTDB 03-RS86 (https://data.ace.uq.edu.au/public/gtdb/data/releases/release86/86.0/) and 04-R89 (https://data.ace.uq.edu.au/public/gtdb/data/releases/release89/89.0/), dbCAN v6 (http://bcb.unl.edu/dbCAN2/download/Databases/), Pfam r31 (ftp://ftp.ebi.ac.uk/pub/databases/Pfam/releases/Pfam31.0/), TIGRFAM v15 (ftp://ftp.jcvi.org/pub/data/TIGRFAMs/) and UniProt UniRef100 (ftp://ftp.uniprot.org/pub/databases/uniprot/previous_releases/release-2017_06/).

Field-specific reporting

Please select the one below that is the best fit for your research. If you are not sure, read the appropriate sections before making your selection.

☒ Life sciences ☐ Behavioural & social sciences ☐ Ecological, evolutionary & environmental sciences

For a reference copy of the document with all sections, see nature.com/documents/nr-reporting-summary-flat.pdf

Life sciences study design

All studies must disclose on these points even when the disclosure is negative.

Sample size	It is unfortunately not possible for us to perform a statistical calculation to estimate the appropriate number of participants required for our proposed study as we have no human fecal microbiota data in hand (necessary for performing a power test). Published studies looking at fecal microbiota vary widely in sample size and do not indicate how the participant numbers were devised. Published studies investigating differences in fecal microbiota between healthy individuals and those with inflammatory bowel disease (IBD) range widely from only 5 healthy controls and 11 IBD patients (Gophna 2006 J Clin Microbiol 44:4136) to 65 healthy controls and 96 IBD patients (Takaishi 2008 Int J Med Microbiol 298:463). A study investigating the fecal microbiota of a family of 8 persons (e.g. shared environment and diet) with that of a cohort of 155 unrelated persons reported that while each member of the family had a microbiota that was “personalised” and distinct from the community, the constituent species of the microbiota were consistent for each person but the abundances of the individual species populations were variable day to day (Schloss 2014 Microbiome 2:25).Furthermore, recently published study investigating microbial imbalance in IBD utilized only 30 individual fecal samples. The study reported IBD patients showed a less diverse gut microbiome compared to healthy individuals (Alam 2020 Gut pathogens 12,1)<br><br>Based on the literature, we feel that 50 controls and 50 COPD patients is a good middle ground between the extremes in sample size. No statistical test carried out. As no study of the gastrointestinal microbiome in COPD had been performed, and no preliminary data was available, sample size was selected based upon a review of the literature in the study.
Data exclusions	No data was excluded.
Replication	Each sample from the study cohort was sequenced using both 16S rRNA gene amplicon and metagenomic sequencing of the same DNA extraction producing overlapping microbiome profiles. Results were then validated using metagenomic sequencing of an independent validation cohort, with 16% of bacterial species identified in the study cohort as significantly altered in association with COPD also identified in the validation cohort.
Randomization	As this was an observational study, there was no assignment of patients. Analyses were adjusted for covariates (age, sex, BMI) using a linear model.
Blinding	Extractions were performed by a technician unfamiliar with the project design and were assigned a de-identified sample code prior to sequencing. Blinding was carried out for extractions and followed up with de identified sample code prior to 16S rRNA sequencing and metagenomics. For process of analysis blinding was done for unsupervised analysis for 16S rRNA sequencing and metagenomics data. For other downstream analysis blinding was not done as knowledge of the sample group was essential to the analysis.<br>For metabolomics samples were assigned a unique identifier associated with source identifier only into the Metabolon LIMS system for extractions. The identifier was tracked for analysis process. For analysis unsupervised method blinding was done and for downstream subsequent analysis blinding was not done as knowledge of the sample group was essential to the analysis.

Reporting for specific materials, systems and methods

We require information from authors about some types of materials, experimental systems and methods used in many studies. Here, indicate whether each material, system or method listed is relevant to your study. If you are not sure if a list item applies to your research, read the appropriate section before selecting a response.

Materials & experimental systems

n/a	Involved in the study
☒	☐ Antibodies
☒	☐ Eukaryotic cell lines
☒	☐ Palaeontology and archaeology
☒	☐ Animals and other organisms
☐	☒ Human research participants
☐	☒ Clinical data
☒	☐ Dual use research of concern

Methods

n/a	Involved in the study
☒	☐ ChIP-seq
☒	☐ Flow cytometry
☒	☐ MRI-based neuroimaging

Human research participants

Policy information about studies involving human research participants

Population characteristics

Twenty-eight COPD patients and 29 healthy controls were recruited from John Hunter Hospital, Belmont District Hospital, Newcastle Community Health Centre, Westlakes Community Health centre and Hunter Medical Research Institute (Newcastle, Australia). Participants were attending pulmonary rehabilitation programmes at these sites, with a defined diagnosis of COPD. COPD participants all were >40 years old and had a previous history of smoking. Healthy controls were adults >40 years old with no history of cardiac or respiratory disease, and with normal lung function measured by spirometry (FEV1/FVC ratio >0.7 and FEV1 >80% predicted). There were a greater percentage of females in the HC group (66%) relative the COPD group (54%), and the mean ages were 60.4 years for the HC group (median 62) and 67 for the COPD group (median 68).

For the independent validation cohort, sixteen COPD patients and twenty two healthy individuals were recruited from the thoracic outpatient clinic at The Prince Charles Hospital (Brisbane Australia) and from the general population, respectively. Patients had a defined diagnosis of COPD. For validation cohort, COPD patients were former smokers of ≥10 years, who are recruited during stability (>4 weeks since an exacerbation). Healthy controls were adults >40 years old with no history of cardiac or respiratory disease. There were a greater percentage of females in the HC group (68.18%) relative the COPD group (43.75%), and the mean ages were 60.72 years for the HC group and 71.44 for the COPD group. Average BMI was 23.15 for the HC group and 29.11 for the COPD group.

Recruitment

For study cohort, twenty-eight COPD patients and 29 healthy controls were recruited from John Hunter Hospital, Belmont District Hospital, Newcastle Community Health Centre, Westlakes Community Centre and Hunter Medical Research Institute (Newcastle, Australia). The participants were approached by the clinical research office. They were invited to take part in the study and provided with an information and consent form. If they consented to participate, they undertook the procedures as outlined in the methods section. With consent, details in regards their disease and relevant clinical history was confirmed from medical records. For healthy controls, the study was advertised to local community groups and volunteers on the Hunter Medical Research Institute registry. If individuals provided consent, they were contacted by the lead researcher who assessed eligibility.

For the validation cohort, sixteen COPD patients and twenty two healthy individuals were recruited from The Prince Charles Hospital outpatient clinics. The eligibility of the subject was assessed by the lead researcher, and confirmed by a respiratory physician, who then referred the patients for the study. The lead researcher then obtained written informed consent from the subject before data or sample collection. For the healthy participants, the study was advertised to local community groups. These participants contacted the lead researcher to participate in the study.

This two-step recruitment process limits the individual bias in the subject recruitment process and impact on the results.

Ethics oversight

Approval was obtained from the Human Ethics Research Committees of the Hunter New England Local Health District (14/08/20/3.02) and the University of Newcastle (H-2015-0006).

For the validation cohort, ethics approval was obtained from The Prince Charles Hospital Human Research Ethics Committee (HREC/18/QPCH/234) and the University of Queensland (2108001673/HREC/18/QPCH/234).For the validation cohort, written and informed consent was obtained before any data or sample collection.

Note that full information on the approval of the study protocol must also be provided in the manuscript.

Clinical data

Policy information about clinical studies

All manuscripts should comply with the ICMJEguidelines for publication of clinical research and a completedCONSORT checklist must be included with all submissions.

Clinical trial registration

ACTRN12614001286639
ACTRN12618001044213 (validation cohort)

Study protocol	Study protocol is available from the corresponding author upon request.
Data collection	Samples and patient characteristics were collected between March 2015 and November 2016 at Hunter Medical Research Institute (HMRI), Newcastle, Australia. Samples were transported to the University of Queensland, Brisbane, Australia for sequencing (16S rRNA gene and metagenomics) or Metabolon Inc., Durham, USA for metabolomics between January 2017 and November 2018. Final analysis of collected data was performed in HMRI and the University of Queensland.<br><br>For the validation cohort, samples and patient characteristics were collected between April 2019 - March 2020 at The Prince Charles Hospital, Brisbane, Australia. Samples were processed as analysis in accordance with the original cohort.
Outcomes	The primary outcome was to identify whether microbiome and metabolome composition differed in COPD patients compared to healthy controls, and was selected as there has been no previous study investigating the composition of the gastrointestinal microbiome in COPD to this date. Microbiome composition was assessed by sequencing (16S rRNA and metagenomic) and metabolome composition assessed using untargeted metabolomics (UPLC/MS/MS), and analysed using PERMANOVA to compare the entire composition of groups rather than limiting comparisons to specific group of microbes and/or metabolites. Secondary outcomes, of identifying key signatures which were correlated with disease severity, were selected to improve the utility of the current study, to inform on which components of the microbiome/metabolome may impact disease severity. Data collected in the primary outcome was correlated with lung function data collected by spirometry and blood counts collected by pathology services.
