# Supplementary material for: Disease-associated gut microbiome and metabolome changes in patients with chronic obstructive pulmonary disease
Source: Nat Commun. 2020 Nov 18;11:5886. doi: 10.1038/s41467-020-19701-0 (PMC7676259; doi:10.1038/s41467-020-19701-0)
Supplement: Supplementary file 4 — Description of Additional Supplementary Files [file 41467_2020_19701_MOESM4_ESM.pdf]

Description of Additional Supplementary Files

Description: Supplementary Data 1-35

Supplementary Data 1. Patient characteristics, group comparisons

Supplementary Data 2. Patient characteristics, medications

Supplementary Data 3. Patient characteristics, blood count

Supplementary Data 4. Patient characteristics, lung function

Supplementary Data 5. Patient characteristics, nutritional data

Supplementary Data 6. Comparison of COPD and healthy gut microbiome via simple and extended adonis model

Supplementary Data 7. Relative abundance of sequence variants identified from 16S rRNA gene sequencing as contributing to separation between COPD and healthy faecal samples

Supplementary Data 8. Comparison of COPD and healthy gut microbiome as measured using metagenomics sequencing via simple and extended adonis model

Supplementary Data 9. Relative abundance of bacterial families significantly differential between COPD and healthy based on metagenomic sequencing of faecal samples

Supplementary Data 10. Genomes identified as significantly differential between COPD and healthy samples assessed using metagenomic sequencing

Supplementary Data 11. Genomes identified as significantly differential between COPD and healthy samples using DESeq2 with inclusion of additional variables

Supplementary Data 12. DESeq2 analysis of read counts from Pfam annotation

Supplementary Data 13. DESeq2 analysis of read counts from TIGRFAM annotation

Supplementary Data 14. DESeq2 analysis of read counts from KEGG annotation

Supplementary Data 15. DESeq2 analysis of read counts from CAZy annotation

Supplementary Data 16. Genome-based read mapping counts using reads annotated with Pfam PF02324

Supplementary Data 17. Genome-based read mapping counts using reads annotated with TIGRFAM TIGR04035

Supplementary Data 18. Genome-based read mapping counts using reads annotated with KO K00689

Supplementary Data 19. Genome-based read mapping counts using reads annotated with CAZy GH70

Supplementary Data 20. Genome-based read mapping counts using reads annotated with KO K12472

Supplementary Data 21. Genome-based read mapping counts using reads annotated with TIGRFAM TIGR04225

Supplementary Data 22. KEGG orthology enrichment analysis

Supplementary Data 23. KEGG module completeness across genomes enriched in COPD or healthy individuals

Supplementary Data 24. Metabolomics raw data

Supplementary Data 25. Comparison of metabolites between COPD and healthy individuals from mixOmics and covariate adjusted linear models

Supplementary Data 26. BLAST analysis of genes of interest within enriched genomes related to enriched metabolites

Supplementary Data 27. Dietary questionnaire responses

Supplementary Data 28. 16S rRNA gene amplicon sequencing read counts

Supplementary Data 29. Metagenomic sequencing data

Supplementary Data 30. Metagenome-assembled genomes recovered

Supplementary Data 31. Patient characteristics, validation cohort

Supplementary Data 32. Genomes identified as significantly differentiating between COPD and healthy samples in the study cohort and replicated in the validation cohort

Supplementary Data 33. Functional domains identified as significantly differentiating between COPD and healthy samples in the study cohort compared to the validation cohort

Supplementary Data 34. P values associated with correlation matrix contained in Figure 3

Supplementary Data 35. P values associated with correlation matrix contained in Figure 5
